# Investigation of Combined Toxic Metals, PFAS, Volatile Organic Compounds, and Essential Elements in Chronic Kidney Disease

**DOI:** 10.3390/jox15060202

**Published:** 2025-12-02

**Authors:** Aderonke Gbemi Adetunji, Emmanuel Obeng-Gyasi

**Affiliations:** 1 Department of Built Environment, North Carolina A&T State University, Greensboro, NC 27411, USA; 2 Environmental Health and Disease Laboratory, North Carolina A&T State University, Greensboro, NC 27411, USA

**Keywords:** environmental pollutants, kidney function, toxic metals, PFAS, mixture analysis

## Abstract

Exposure to environmental pollutants, including toxic metals, volatile organic compounds (VOCs), and per- and polyfluoroalkyl substances (PFAS), has been increasingly linked to impaired kidney function. However, the combined effects of these exposures, along with essential elements, on kidney health remain poorly understood. This study aimed to evaluate the independent and cumulative or mixture effects of toxic metals (cadmium, lead, and mercury), essential elements (iron, manganese, and selenium), PFAS (PFOA and PFOS), and VOCs (m-/p-xylene and o-xylene) on kidney function as measured by estimated glomerular filtration rate (eGFR). Using data from the National Health and Nutrition Examination Survey (NHANES), we applied multiple imputation to address missing data and implemented statistical techniques, including Bayesian Kernel Machine Regression (BKMR), quantile g-computation, and Weighted Quantile Sum Regression (WQSR) to assess complex exposure–response relationships, including non-linear, potential synergistic, and antagonistic effects. The results indicated that several exposures were correlated, particularly o-xylene with m-/p-xylene (r = 0.77), Cd with Pb (r = 0.46), and PFOS with PFOA (r = 0.61). eGFR was negatively associated with Pb, PFOS, PFOA, and Hg. In the BKMR analysis, overall posterior inclusion probabilities (PIPs) highlighted PFOS, Cd, Se, Mn, and Fe as the most influential exposures. Quantile g-computation highlighted Cd and Mn as major contributors, while WQSR modeling confirmed Mn as a key contributor. The findings underscore the importance of considering complex interactions in environmental exposure assessments. While essential elements may offer protective effects, toxic metals, PFAS, and VOCs remain critical contributors to kidney dysfunction. These insights highlight the need for integrative risk assessment approaches and public health strategies aimed at mitigating harmful exposures while promoting optimal nutrient balance.

## 1. Introduction

### Overview of Chronic Kidney Disease

Chronic kidney disease (CKD) is a critical global health issue, impacting millions of individuals around the world and placing a heavy burden on healthcare systems in both developed and developing countries, with far-reaching implications for morbidity and mortality [1,2]. It was estimated that about 13.4% of the global population is affected by CKD, which translates to roughly 850 million individuals worldwide [3,4]. This figure underscores the escalating burden of kidney disease across diverse populations. In the United States, the data reveals a similarly alarming pattern. The Centers for Disease Control and Prevention (CDC) estimates that CKD affects around 35.5 million adults, approximately 15% of the adult population, or more than one in seven individuals [5]. The condition is slightly more common in females (14%) compared to males (12%). Prevalence is notably higher among those aged 65 and older (34%), as opposed to 12% in the 45–64 age group and 6% in the 18–44 age group. Additionally, non-Hispanic Black adults experience a higher prevalence (20%) than non-Hispanic Asian (14%) or non-Hispanic white adults (12%), while about 14% of Hispanic adults are affected. Alarmingly, up to 90% of adults with CKD are unaware of their condition because the early stages often present no noticeable symptoms [5].

CKD has well-recognized primary causes, including diabetes mellitus, hypertension, and glomerulonephritis. In addition to these established etiologies, increasing attention has turned toward environmental exposures as secondary or contributory risk factors in CKD pathogenesis.

The kidneys perform vital functions essential for maintaining overall health, including regulating fluid and electrolyte balance through filtration and excretion, removing waste products to preserve homeostasis, and controlling blood pressure by releasing renin, an enzyme involved in the renin–angiotensin–aldosterone system that manages blood volume and vascular tone [6]. CKD is characterized by a gradual, often irreversible loss of kidney function that can progress to end-stage renal disease (ESRD), requiring dialysis or transplantation [7,8]. As kidney function declines, toxins and fluid accumulate in the body, increasing the risk of hypertension, cardiovascular disease, stroke, and premature death [7,8].

The most common causes of CKD are diabetes mellitus and hypertension, together accounting for about 75% of cases globally. Diabetes damages renal blood vessels and glomeruli through hyperglycemia-induced oxidative stress, while hypertension causes vascular injury from elevated intraglomerular pressure [9]. Other significant causes include glomerulonephritis, polycystic kidney disease, prolonged use of nephrotoxic medications, such as NSAIDs and certain antibiotics, as well as lifestyle factors like obesity, smoking, and environmental toxin exposure [10,11,12,13].

In some regions, particularly Central America, a distinct form of chronic kidney disease known as CKD of nontraditional etiology (CKDnT), or Mesoamerican nephropathy, affects mainly young male agricultural workers in hot, low-altitude areas. Unlike typical CKD, it is not linked to diabetes or hypertension but is thought to result from repeated heat stress, dehydration, exposure to pesticides and heavy metals, and other metabolic disturbances, though its exact cause remains multifactorial and not fully understood [14,15].

CKD is closely linked to the body’s balance of environmental exposures and essential elements. Toxic metals such as lead (Pb), cadmium (Cd), chromium (Cr), and mercury (Hg) can accumulate over time, disrupting kidney function by damaging cellular organelles involved in metabolism, detoxification, and tissue repair [16]. Environmental exposure to these metals, commonly found in contaminated water and food, has been associated with reduced kidney function and a higher risk of CKD [17,18,19]. In a cross-sectional study analyzing exposure to low levels of heavy metals and CKD among the US adult population, using data from the National Health and Nutrition Examination Survey (NHANES) between 1999 and 2020, researchers found that low blood concentrations of Cd and Pb were associated with increased odds of CKD, whereas low blood concentrations of Hg were linked to decreased odds of CKD [20]. In addition, per- and polyfluoroalkyl substances (PFAS), including Perfluorooctanoic acid (PFOA) and perfluorooctane sulfonate (PFOS), two of the most extensively studied per- and polyfluoroalkyl substances (PFAS), have been increasingly implicated in the pathogenesis of CKD [21]. These persistent environmental pollutants are nephrotoxic due to their bioaccumulative nature and long biological half-lives [22]. Elevated serum levels of PFOA and PFOS have been associated with reduced glomerular filtration rate (GFR), increased serum creatinine levels, and overall renal impairment [23]. Mechanistically, PFAS may promote oxidative stress, mitochondrial dysfunction, and inflammation in renal tissue, leading to tubulointerstitial damage and fibrosis [24]. while also disrupting peroxisome proliferator-activated receptor (PPAR) signaling, a key pathway in lipid metabolism and renal homeostasis [25].

Volatile organic compounds (VOCs) are a diverse group of gaseous chemicals widely present in the environment and commonly used in residential, commercial, and industrial applications, such as solvents, degreasers, and cleaning agents [26]. They can be anthropogenic—emitted from industrial processes, vehicle exhaust, and consumer products—or biogenic, naturally released by plants, particularly in tropical regions [27,28]. Urban areas typically exhibit higher VOC concentrations due to traffic emissions, fuel combustion, and industrial activities like petrochemical manufacturing, dry cleaning, and painting. VOCs are also found in everyday products such as paints, varnishes, glues, and waxes [29]. Because VOCs are highly reactive, they quickly undergo physical and chemical transformations upon release into the atmosphere, generating various secondary organic and inorganic pollutants. Exposure to VOCs has been associated with various diseases; however, current studies are limited, often examining individual compounds rather than assessing cumulative or combined exposure effects [30,31].

In contrast to toxic metals, PFAS and VOCs, essential elements play critical roles in various physiological processes, such as antioxidant defense, enzyme activity, and the regulation of kidney metabolism. Deficiencies in these essential elements can negatively affect kidney function and accelerate health deterioration in CKD patients [32,33,34]. Additionally, insufficient levels or an imbalance of essential elements can worsen the harmful effects of toxic metal exposure, further compromising kidney health [35,36]. Plasma Mn, Fe and Zn levels have been shown to protect against CKD in elderly populations [37].

Existing studies have examined the associations between exposure to toxic metals, volatile organic compounds (VOCs), per- and polyfluoroalkyl substances (PFAS), and essential elements with CKD individually. However, the potential combined or synergistic effects of these exposures on kidney health remain largely unexplored. The complex interactions among toxic metals, VOCs, PFAS, and essential elements within the renal system may significantly influence the onset and progression of CKD. Understanding these interactions is essential for developing effective prevention and management strategies. Reducing exposure to harmful substances while maintaining adequate levels of essential elements through diet or supplementation may help mitigate kidney damage and slow disease progression. This study aims to evaluate both the individual and joint effects of toxic metals (Pb, Cd, Hg), PFAS (PFOA, PFOS), VOCs (o-xylene, m-/p-xylene), and essential elements (Fe, Mn, Se) on CKD risk and kidney function—measured by estimated glomerular filtration rate (eGFR)—using National Health and Nutrition Examination Survey (NHANES) data.

## 2. Materials and Methods

### 2.1. Participant Selection and Study Design

The present analysis utilized data from the 2017–2018 cycle of the National Health and Nutrition Examination Survey (NHANES). NHANES is a nationwide, population-based survey designed to assess health, nutrition, and environmental exposures among noninstitutionalized U.S. residents. The program, administered by the National Center for Health Statistics (NCHS) at the Centers for Disease Control and Prevention (CDC), collects data in two-year intervals using a complex, multistage probability sampling design that ensures representation across demographic and geographic groups. During the 2017–2018 survey period, a total of 16,211 participants were selected from 30 primary sampling sites across the United States and the District of Columbia. Of those selected, 9254 completed the interview and 8704 were examined. All participants provided written informed consent before taking part in the study. Trained personnel conducted structured interviews and comprehensive physical examinations. Blood specimens were obtained and analyzed in certified laboratories following standardized NHANES protocols. The National Center for Health Statistics (NCHS) Institutional Review Board approved all study procedures to ensure adherence to ethical and regulatory standards. Demographic characteristics such as age, sex, and race/ethnicity were gathered during household interviews using a Computer-Assisted Personal Interviewing (CAPI) system, designed to enhance data accuracy and minimize interviewer bias. To promote inclusivity and data quality, interviews were conducted in multiple languages according to participant preference.

### 2.2. Glomerular Filtration Rate, Essential Elements, Metals, PFAS, and Volatile Organic Compounds Quantification

#### 2.2.1. eGFR Calculation

Renal function was quantified by estimating the glomerular filtration rate (eGFR) using the Modification of Diet in Renal Disease (MDRD) equation. This formula is an established and validated approach in epidemiologic and clinical research for approximating the filtration capacity of the kidneys based on serum creatinine concentration, age, sex, and race.

Kidney filtration efficiency was estimated using the Modification of Diet in Renal Disease (MDRD) equation developed by Levey and colleagues (1999). The MDRD formula is as follows:

$${\mathrm{eGFR}\;(\mathrm{mL}/\min/1.73\;m}^{2}\;=\;175\times (\mathrm{Scr}{)}^{-1.154}\times (\mathrm{Age}{)}^{-0.203}\times (0.742\;\mathrm{if}\;\mathrm{female})\times (1.210\;\mathrm{if}\;\mathrm{Black})$$

where Scr represents the serum creatinine concentration (mg/dL) and Age is measured in years. Sex- and race-specific coefficients were applied as recommended in the original MDRD study to improve accuracy across demographic subgroups.

#### 2.2.2. Laboratory Analyses

All biomarker measurements followed NHANES 2017–2018 laboratory protocols. Whole blood concentrations of toxic metals including Pb), Cd, and Hg, as well as essential trace elements such as Mn) and Se, were quantified using inductively coupled plasma mass spectrometry with a dynamic reaction cell (ICP-DRC-MS). Blood samples (0.25 mL), collected in metal-free EDTA tubes, were diluted 1:50 with an alkaline reagent containing tetramethylammonium hydroxide, Triton X-100, and internal standards (rhodium, iridium, and tellurium), and analyzed using the PerkinElmer ELAN DRC II ICP-MS system (PerkinElmer, Norwalk, CT, USA). Serum iron (Fe) was measured using a Ferrozine-based colorimetric assay on the Roche Cobas 6000 analyzer (c501 module; Roche Diagnostics, Mannheim, Germany), which involves acid and detergent-mediated release of Fe^3+^ from transferrin, reduction to Fe^2+^ by ascorbate, and formation of a Ferrozine-Fe^2+^ complex for photometric detection. PFAS, including PFOA and PFOS, were assessed in serum using automated online solid-phase extraction combined with high-performance liquid chromatography–tandem mass spectrometry (on-line SPE HPLC–MS/MS). Analyses were conducted on a Spark Holland Symbiosis SPE system (Spark Holland, Emmen, Netherlands) coupled with a Sciex API 5500 or 6500 triple quadrupole mass spectrometer (SCIEX, Framingham, MA, USA) operating in negative electrospray ionization mode. Separation was achieved on a C18 reversed-phase column using ammonium acetate (20 mM, pH 4) and acetonitrile as the mobile phase. Detection was performed using multiple reaction monitoring (MRM), and isotopically labeled internal standards were used for quantification. Selected volatile organic compounds (VOCs), specifically o-xylene and m-/p-xylene, were measured in whole blood using headspace solid-phase microextraction (SPME) with a 75 µm Carboxen/PDMS fiber (Supelco, Bellefonte, PA, USA) and a CTC PAL autosampler (CTC Analytics AG, Zwingen, Switzerland), coupled to a gas chromatograph–mass spectrometer (Agilent Technologies, Santa Clara, CA, USA) equipped with a DB-VRX capillary column (Agilent Technologies, Santa Clara, CA, USA). Isotope-dilution calibration was used for quantification. Blood samples (10 mL) were incubated at 40 °C, and a 75 µm Carboxen/PDMS fiber (Supelco) was used for headspace extraction with a CTC PAL autosampler. VOCs were thermally desorbed at 250 °C into the gas chromatograph inlet and separated on a 40 m × 0.18 mm internal diameter DB-VRX capillary column with a 1.0 µm film thickness (Agilent Technologies, Santa Clara, CA, USA). Quantification was performed using a quadrupole mass detector in selected-ion monitoring mode. For all analytes, values below the limit of detection (LOD) were substituted with LOD divided by the square root of 2 (LOD/√2) to minimize estimation bias [38].

### 2.3. Statistical Analysis

#### 2.3.1. Handling of Missing Data Using Multiple Imputation by Chained Equation (MICE)

The missing data in our dataset was handled using MICE [39]. This method assumes that data are missing at random (MAR) and fills in missing values through an iterative process that uses conditional models, maintaining relationships among variables and minimizing bias. Our dataset includes outcome variable (eGFR), exposure variables (VOCs, PFAS, heavy metals), essential elements and covariates. Patterns of missingness were assessed using md.pattern(). Continuous variables were imputed with predictive mean matching (pmm), while binary variables were imputed using logistic regression with bootstrapping (“logreg.boot”). The imputation was performed with 20 datasets (m = 20), 20 iterations (maxit = 20), and a random seed of 500 to ensure reproducibility. Convergence was evaluated through diagnostic plots (plot(), stripplot(), and densityplot()), which confirm stable and consistent imputation across iterations. The complete dataset was generated using the complete() function and regression analyses were pooled using Rubin’s rules with the pool() function.

#### 2.3.2. Descriptive Statistics

We explored descriptive statistics in summarizing the key variables in the dataset, which include age, sex, and ethnicity.

#### 2.3.3. Bayesian Kernel Machine Regression (BKMR)

To evaluate mixture effects, a BKMR framework was applied to jointly examine associations between essential elements, toxic metals, and cardiovascular outcome measures. This semi-parametric Bayesian approach allows for the estimation of non-linear and interactive exposure–response relationships while accounting for potential correlations among multiple co-exposures. BKMR is widely utilized because it effectively models non-linear and non-additive relationships, enabling accurate assessment of the joint effects of multiple contaminants. This method is particularly effective at identifying complex interactions and dependencies within data, allowing for a detailed and accurate assessment of the collective influence of exposures on health outcomes [40,41].

The model used in our study is as follows:

g(*µ_i_*) = *h*(*z_i_*_1_, …, *z_iM_*) + *β*X*_i_*; *i* = 1, …, n (1)

where g is the monotonic link function, *µ_i_* = *E*(*Yi*), *h* is the flexible kernel function of exposures *z_i_*_1_, …, *z_iM_* with x being the vector of covariates with a linear association with eGFR, and *β* represents a vector of associated coefficients.

In the BKMR model, the predictors (Z) represent exposure variables, while the exposure–response relationship is captured by the function *h*(.). BKMR employs multiple representations to investigate complex patterns of association and interaction between exposure variables, and cardiovascular disease risk. This modeling approach allows exploration of both individual and combined effects, identifying univariate and bivariate relationships, as well as potential interactions among variables [41,42]. It effectively captures non-linear relationships and dependencies, providing a thorough assessment of how these exposures collectively influence health outcomes.

#### 2.3.4. Weighted Quantile Sum Regression (WQSR)

WQSR was employed to quantify the collective association of correlated exposures with the outcome of interest. This mixture modeling technique is particularly suited for high-dimensional data characterized by multicollinearity among predictors. The procedure involves two sequential steps: first, a training stage in which component-specific weights are derived to represent each exposure’s relative contribution to the mixture index; and second, a validation stage, during which the weighted index is incorporated into a regression framework to estimate the overall mixture effect [43]. The WQSR model is represented as follows:

(2) $$g\left(\mu \right)\;=\;\;\beta_{0}+\beta 1(\sum_{i=1}^{c}w_{i}q_{i})\;+z’\varphi$$

In the WQSR framework, *g*(*μ*) denotes the link function corresponding to the model’s specified distribution, where μ represents the expected value of the response variable. Each exposure variable was converted into quantiles, represented as qi for the *i*th component, and *w_i_* indicates the estimated weight obtained during model fitting. The weight *w_i_* expresses the relative contribution of the *i*th exposure to the overall WQSR index. The vector *z*′ comprises the covariates, with ϕ\phi representing their associated parameters. The term $\sum_{i=1}^{c}w_{i}q_{i}^{q}$ defines the index that combines the mixture components’ contributions by assigning weights to each.

To develop the WQSR model, the dataset was randomly divided into two subsets: a training portion used to derive the mixture weights and a validation portion used to evaluate the significance of the constructed WQSR index. Weight estimation was performed through a bootstrap resampling procedure, with the constraint that all weights lie between zero and one and collectively sum to unity, such that $\sum_{i=1}^{c}w_{i}=1$ and 0 ≤ *w_i_* ≤ 1 for all *i*. During every bootstrap iteration, a new resampled dataset was created by randomly drawing observations with replacement from the original training subset. The model parameters, represented as *θ* = (*β*_0_, *β*_1_, *w*_1_, …, *w_c_*, φ), were estimated through an optimization algorithm, using the log-likelihood function as the objective criterion.

(3) $$\hat{\theta }WQS=argmax\theta \left[l\left(\theta ;y\right)+\lambda \;\left(\sum_{i=1}^{c}w_{i}-1\right)\;\right]$$

Within this optimization framework, *l*(*θ*; *y*) denotes the log-likelihood function, and *λ* is the Lagrange multiplier introduced to impose the normalization constraint, thereby ensuring that the estimated weights satisfy the condition ∑*w_i_* = 1. In addition, an inequality constraint was imposed to restrict the estimated weights to the permissible interval 0 ≤ *w_i_* ≤ 1, thereby maintaining numerical stability and interpretability of the mixture index. Once the weights are estimated, they are used to compute the regression coefficients at each step of the ensemble procedure. After completing the bootstrap ensemble, the weights are averaged for all bootstrap samples to produce the ultimate WQSR index.

(4) $$WQS=\sum_{i=1}^{c}{\bar{w}}_{i}q_{i}$$

where

(5) $${\bar{w}}_{i}=\frac{1}{\sum_{b=1}^{B}f\left(\beta_{i}\left(b\right)\right)}\sum_{b=1}^{B}w_{i}\left(b\right)f\left(\beta_{i}\left(b\right)\right)\;and\;f\left(\beta_{i}\left(b\right)\right)$$

This function acts as the signal estimator within the model. The exposure-specific weights were first derived from the training subset and subsequently applied to the validation subset to construct the WQSR index, representing the overall mixture effect. This index is subsequently used in a standard generalized linear model to evaluate the relationship between the mixture and the health outcome, as described below:

(6) $$g\left(\mu \right)\;=\;\;\beta_{0}+\beta 1\;WQS+z’\varphi$$

The model constrained the relationship between the outcome variable and the WQSR index to a positive monotonic direction. This specification ensured that the analysis focused on mixture effects exhibiting consistent positive associations with the outcome. By limiting estimation to the positive constraint, the model identified exposures contributing collectively to an increase in the outcome measure, providing a clear interpretation of the direction and magnitude of the overall mixture effect. This modeling strategy minimized the potential for a reversal paradox, which can arise in the presence of strong intercorrelations among exposure variables. The use of a bootstrap resampling procedure further enhanced the robustness of weight estimation and facilitated the identification of influential components within the mixture. After fitting the final model, the statistical significance of *β*_1_ was evaluated to assess whether the WQSR index demonstrated a meaningful association with the outcome variable. When *β*_1_ was statistically different from zero, the individual weights were examined, with larger weights indicating the primary exposure components driving the observed association. In this analysis, the WQSR index representing exposure to heavy metals and essential elements was derived from the quartile distributions of blood concentrations for each metal and element. The data was divided into a training set (60%) and a testing set (40%). The WQSR model also adjusted for potential confounders, including age, sex, race/ethnicity, BMI, income, smoking status, and alcohol consumption.

#### 2.3.5. Quantile G-Computation

Quantile g-computation enhances mixture analysis by accommodating both non-linear and nonadditive exposure–response relationships. The method begins by transforming each exposure variable (Xj) into its corresponding quantized form (Xqj), thereby standardizing scales across components. A generalized linear modeling framework is then applied to estimate the combined association between the quantized exposures and the health outcome, allowing for flexible inference on the joint effect of the mixture:

(7) $$Y_{i}\;=\;\beta_{0}+\sum_{j=1}^{d}\beta_{j}X_{\;ji}^{\;q}+\beta_{j}Z+\varepsilon_{i}$$

where $Y_{i}$ represents the outcome for the *i*-th participant, $\beta_{0}$ is the intercept, and $X_{ji}^{\left(q\right)}$ denotes the quantized version of the *j*-th exposure variable for individual *i*. The coefficients $\beta_{j}$ quantify the effect of each exposure on the outcome. The term $\beta Z_{i}$ accounts for the influence of covariates such as demographic, socioeconomic, or behavioral characteristics, while $\varepsilon_{i}$ captures random error and unexplained variation.

Under the assumption of directional homogeneity, the overall mixture effect parameter ψ is defined as follows:

(8) $$\psi \;=\;\sum_{j=1}^{d},\beta_{j}$$

which represents the combined contribution of all exposures in the same direction. The relative importance of each exposure is expressed as a weight $w_{k}$, calculated as follows:

(9) $$w_{k}\;=\;\frac{\beta_{k}}{\sum_{j=1}^{d}\cdot \beta_{j}}$$

ensuring that the weights sum to unity, $\sum w_{k}\;=\;1$.

This modeling framework is highly adaptable and suitable for a range of exposure types and health outcomes. In the present analysis, quantile g-computation was implemented to examine the combined and individual contributions of essential elements, toxic metals, PFAS, and VOCs to variations in eGFR. The model further quantified the expected change in eGFR associated with a concurrent one-quantile increase across all exposures within the mixture [27].

Although the NHANES dataset employs a complex, multistage sampling design with survey weights, strata, and primary sampling units (PSUs), the advanced mixture modeling frameworks used in this study, Bayesian Kernel Machine Regression (BKMR), WQSR, and quantile g-computation, do not currently support the direct incorporation of these design elements. This limitation arises because BKMR relies on a Gaussian process kernel that models joint exposure–response relationships through covariance structures rather than weighted likelihoods, while WQSR and quantile g-computation use resampling and quantile transformation procedures that are not compatible with survey weighting. Consequently, these models were estimated without weights, consistent with standard practice in environmental mixture epidemiology. Results should therefore be interpreted as representative of the NHANES sampled population rather than as nationally weighted estimates.

All analyses were performed in R (version 4.2.1; R Foundation for Statistical Computing, Vienna, Austria), using a significance level of 0.05 for non-Bayesian analysis.

## 3. Results

### 3.1. Demographic Overview of Participants by Age, Sex, and Ethnicity

Descriptive statistics and participant characteristics are detailed in Table 1. The demographic profile revealed differences in age distributions when stratified by biological sex and population subgroup. Specifically, the average age among males was 37 years, compared with 39 years among females. Ethnic subgroups also demonstrated variation, with non-Hispanic whites comprising an older demographic profile, whereas Mexican Americans represented a comparatively younger group [44,45].

### 3.2. Pearson Correlation Matrix of Variables of Exposure and Outcome Variables Interest

**Figure 1:**
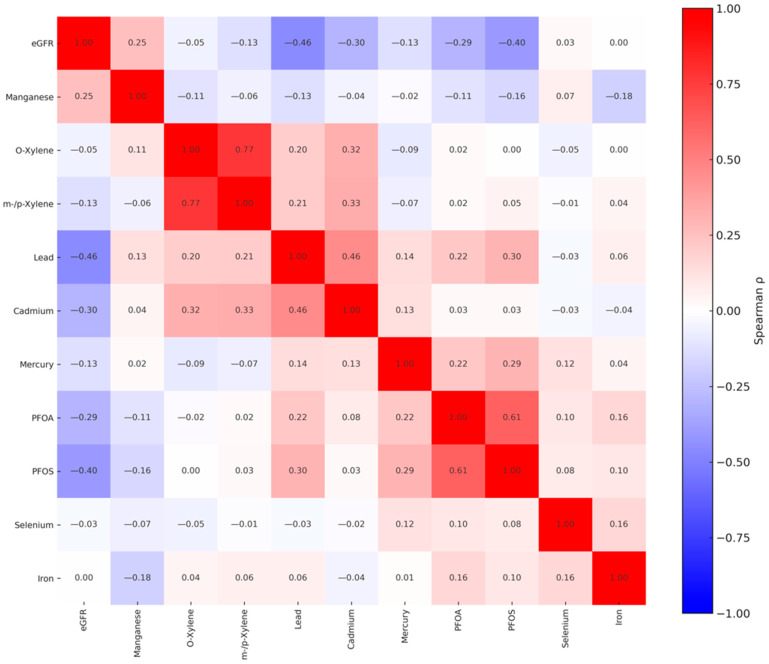
Spearman rank correlations among exposure and outcome variables included in the study. PFOS: perfluorooctanesulfonic acid, PFOA: perfluorooctanoic acid, eGFR: estimated glomerular filtration rate.

Figure 1 represents a correlation matrix of eGFR and environmental exposure to metals, VOCs, PFAS compounds, and essential elements. The analysis indicated that correlation patterns within these groups differed in magnitude and direction. The matrix illustrates Pearson correlation coefficients, with values ranging from −1 (perfect negative correlation) to 1 (perfect positive correlation). Strong positive correlations were observed between several chemical exposures, notably between o-xylene and m-/p-xylene (r = 0.77), Cd and Pb (r = 0.46), and PFOS and PFOA (r = 0.61). In contrast, eGFR was negatively correlated with Pb (r = −0.46) and PFOS (r = −0.40), PFOA (r = −0.29), and Hg (r = −0.13).

### 3.3. BKMR Analysis

#### 3.3.1. Posterior Inclusion Probabilities (PIPs)

Table 2 presents the PIPs estimated for each exposure in relation to the eGFR outcome. The PIP quantifies the likelihood that a given exposure meaningfully contributes to the variability in the modeled health endpoint. A PIP value closer to 1 indicates a higher likelihood that the variable is an important predictor in the model. In this analysis, PFOS, Cd, Se, Mn, and Fe all had PIPs of 1.0000, highlighting them as the most influential exposures. O-xylene (PIP = 0.7224), m-/p-xylene (0.5392), and Pb (0.4880) demonstrated moderate importance, while PFOA (0.2840) and Hg (0.0904) had lower inclusion probabilities, suggesting less importance with the outcome when considering the full exposure mixture.

#### 3.3.2. Hierarchical BKMR Results for Posterior Inclusion Probabilities

HierarchicalBKMR (Table 3) extends standard BKMR by incorporating a grouped structure of exposures. Rather than treating all exposures as completely independent, hierarchical BKMR allows exposures to be organized into groups (e.g., metals, VOCs, PFAS, essential elements). This structure enables the model to first estimate the overall importance of an entire exposure group, and then, conditional on group inclusion, to evaluate the contribution of specific exposures within that group.

All four exposure groups had Group PIPs close to or equal to 1.000, indicating strong overall relevance to the outcome. Within Group 1 (VOCs), o-xylene had a higher Conditional PIP (0.82) than m-/p-xylene (0.18), suggesting it was the primary driver of the group’s importance. In Group 2 (PFAS), PFOS had a Conditional PIP of 1.000, while PFOA had a value of 0.000, indicating PFOS accounted entirely for the group’s influence. Similarly, in Group 3 (metals), Cd was the dominant contributor (Conditional PIP = 1.000), with Pb and Hg showing no conditional contribution. In Group 4 (essential elements), Mn emerged as the key driver (Conditional PIP = 1.000), while Se and Fe had no conditional importance. These results suggest that while multiple exposure groups are associated with the outcome, only specific chemicals within each group are likely responsible for the observed effects.

#### 3.3.3. Univariate Association of Toxic Exposures Metals, VOCs, PFAS Compounds, and Essential Elements with eGFR

**Figure 2:**
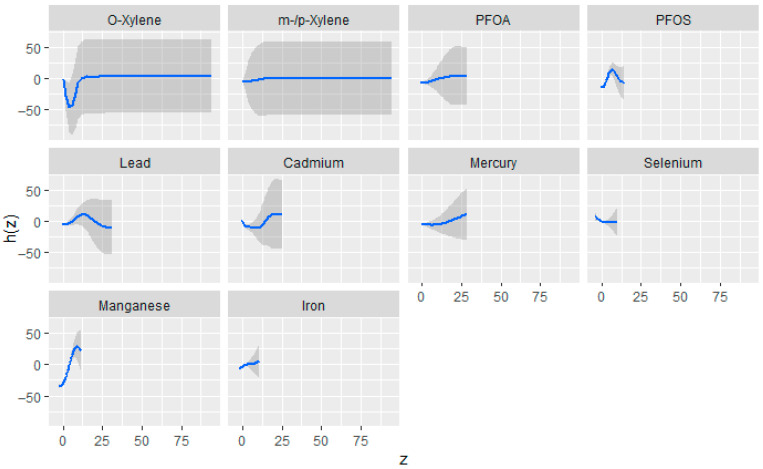
Estimated univariate exposure–response relationships and corresponding 95% credible intervals from the BKMR model, depicting the association between individual exposures and eGFR while all other exposures are maintained at their median levels. Adjusted for diabetes, alcohol use, smoking, income, gender, education, age, BMI, and race/ethnicity. PFOS: perfluorooctanesulfonic acid, PFOA: perfluorooctanoic acid, eGFR: estimated glomerular filtration rate.

Figure 2 illustrates the univariate exposure–response relationships estimated using the BKMR model. Each curve represents the association between an individual exposure and the outcome while holding all other exposures constant at their median values, with covariates statistically controlled. The shaded gray regions denote 95% credible intervals. The vertical axis depicts the estimated exposure–response function h(z), and the horizontal axis corresponds to the quantile range of each exposure variable. In this figure, the exposures demonstrated evidence of non-linear associations. Mn exhibited a strong positive association across increasing quantiles, while Cd showed mild increases in h(z), indicating potential positive associations with the outcome. Pb and PFOS had an inverse ‘U’ shape. For O-xylene, a non-linear ‘U’-shaped association which became fat with increased exposure, whereas PFOA and m-/p-xylene displayed relatively flat curves, suggesting limited association. Fe, Se, and Hg showed flat relationships.

#### 3.3.4. Bivariate Exposure–Response Relationships Between Toxic Exposures and eGFR

**Figure 3:**
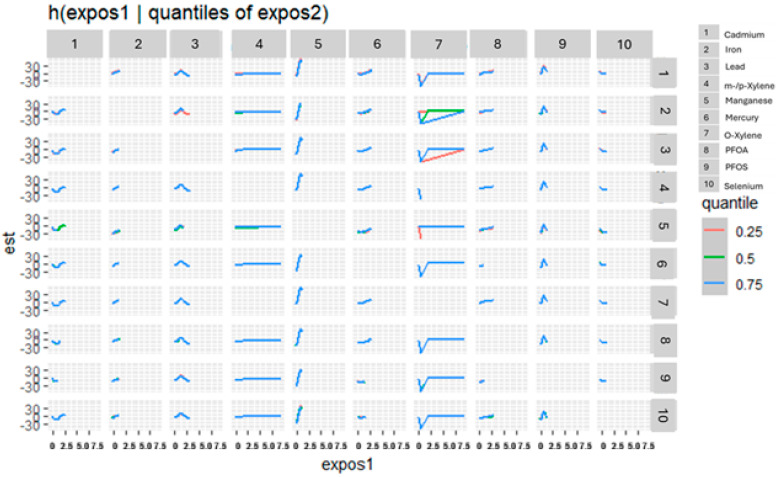
Bivariate exposure–response functions depicting the joint associations of toxic metals, VOCs, PFAS, and essential elements with eGFR. Exposure 1 increases along the *x*-axis, while the second exposure is fixed at the 25th, 50th, and 75th quantiles. Adjusted for diabetes, alcohol use, smoking, income, gender, education, age, BMI and race/ethnicity. PFOS: perfluorooctanesulfonic acid, PFOA: perfluorooctanoic acid, eGFR: estimated glomerular filtration rate.

Figure 3 presents the bivariate exposure–response surfaces derived from the BKMR model, depicting the joint associations of metals, volatile organic compounds (VOCs), per- and polyfluoroalkyl substances (PFAS), and essential elements with estimated glomerular filtration rate (eGFR). For each exposure pair, the first exposure varies across its observed range, while the second exposure is fixed sequentially at the 25th (red), 50th (green), and 75th (blue) percentiles. All remaining exposures were held constant at their median concentrations. The models were statistically adjusted for relevant covariates to control for potential confounding. The horizontal axis (“expos1”) represents the varying exposure, the vertical axis (“est”) corresponds to the estimated effect on eGFR, and the grid of rows and columns displays the paired exposures evaluated in the analysis.

#### 3.3.5. Single-Variable Effects of Exposures on eGFR

**Figure 4:**
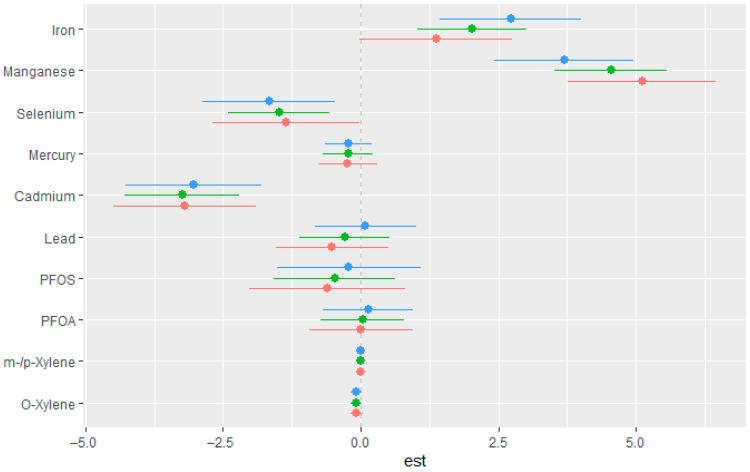
Estimated single-exposure effects with 95% credible intervals showing the change in eGFR associated with increasing each exposure from the 25th to the 75th percentile. All other mixture components were fixed at the 25th (red), 50th (green), and 75th (blue) percentiles. Adjusted for diabetes, alcohol use, smoking, income, gender, education, age, BMI and race/ethnicity. PFOS: perfluorooctanesulfonic acid, PFOA: perfluorooctanoic acid, eGFR: estimated glomerular filtration rate.

This component of the analysis focused on evaluating the marginal influence of each exposure on eGFR across varying concentration levels. For each chemical, point estimates and their corresponding 95% credible intervals were obtained at the 25th (red), 50th (green), and 75th (blue) percentiles of the interacting variable, with all other exposures fixed at median values. Examining these single-variable effects provided clearer insight into how individual exposures contribute to variability in eGFR in the context of other mixture components. As seen in Figure 4, Mn and Fe showed strong positive associations with the outcome, particularly at all quantiles, while Se, Cd, Hg, and PFOS demonstrated negative associations across quantiles. The effects of Pb and PFAO showed mixed effects with overlapping intervals, suggesting weak or uncertain associations. O-xylene and m-/p-xylene had estimates close to zero across all strata, indicating minimal impact in this context.

#### 3.3.6. Single-Variable Interaction Terms of Exposures

**Figure 5:**
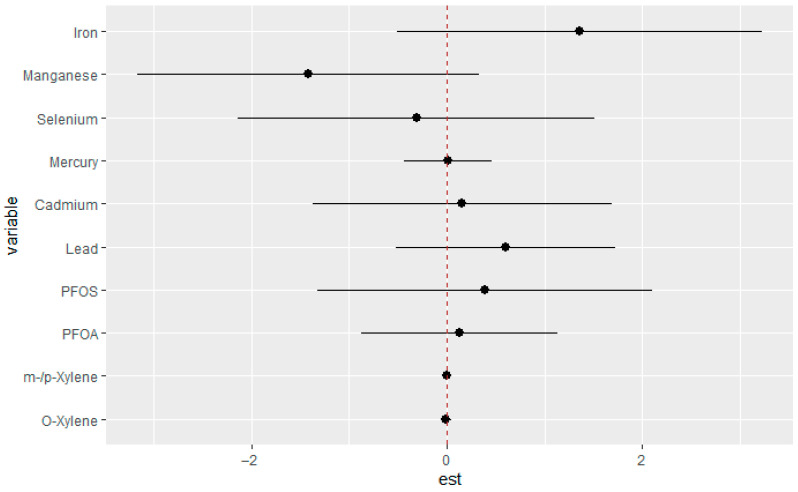
Interaction effects were estimated for each exposure individually, contrasting predicted outcomes when all other exposures were fixed at their 75th and 25th percentile concentrations. Adjusted for diabetes, alcohol use, smoking, income, gender, education, age, BMI, and race/ethnicity. PFOS: perfluorooctanesulfonic acid, PFOA: perfluorooctanoic acid, eGFR: estimated glomerular filtration rate.

The single-variable interaction analysis evaluated the effect of each individual exposure while holding all other exposures constant, comparing estimates when the remaining exposures were fixed at the 75th versus the 25th percentile. Among the analyzed exposures, Fe and Mn demonstrated the strongest interaction patterns. The corresponding results are presented in Figure 5.

#### 3.3.7. Overall Exposure Effect of All Exposures on eGFR

**Figure 6:**
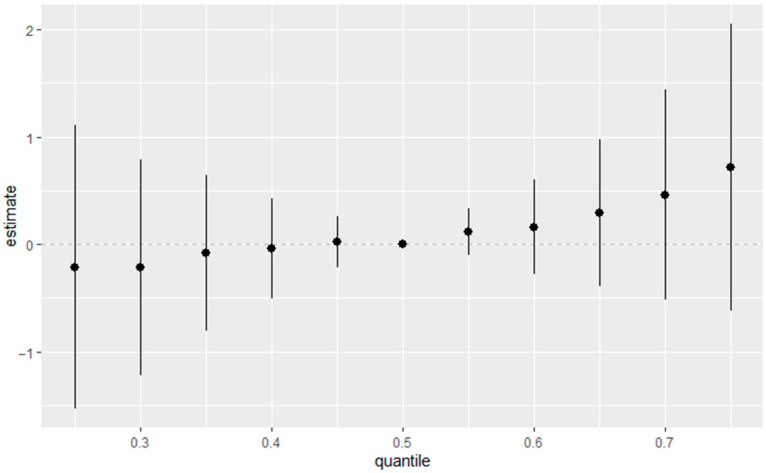
Overall estimated health effects of the combined exposure mixture, calculated by comparing the BKMR function h(z) when all predictors are set to a specific percentile against the value of h(z) when all predictors are fixed at the 50th percentile (median). Adjusted for diabetes, alcohol use, smoking, income, gender, education, age, BMI and race/ethnicity. PFOS: perfluorooctanesulfonic acid, PFOA: perfluorooctanoic acid, eGFR: estimated glomerular filtration rate.

Figure 6 depicts the overall mixture–response relationship between combined exposure quantiles and estimated eGFR as modeled by BKMR. The figure summarizes the cumulative effect of all exposures jointly, illustrating how incremental increases in the exposure mixture correspond to changes in eGFR. In this analysis, all exposures were fixed at quantiles ranging from the 25th to the 75th percentile, using the 50th percentile (median) as the reference. The horizontal axis displays the quantiles of the aggregated exposure distribution, while the vertical axis presents the estimated effect on eGFR with associated 95% credible intervals.

### 3.4. Quantile G-Computation Results

**Figure 7:**
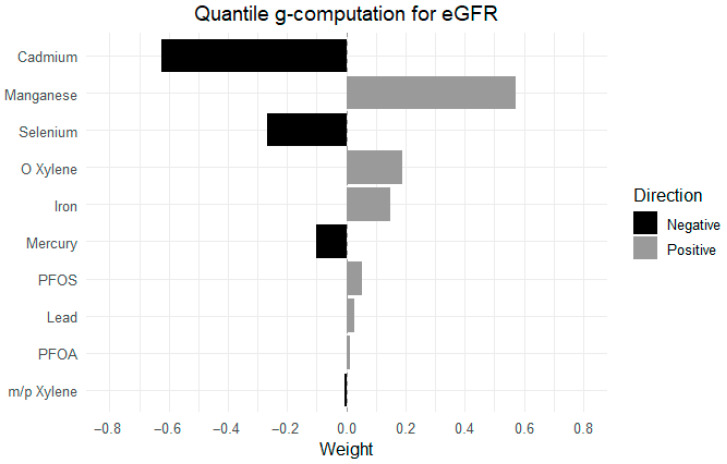
Relative weights of individual exposures in their association with eGFR, estimated using the quantile g-computation model. Adjusted for diabetes, alcohol use, smoking, income, gender, education, age, BMI and race/ethnicity. PFOS: perfluorooctanesulfonic acid, PFOA: perfluorooctanoic acid, eGFR: estimated glomerular filtration rate.

Figure 7 presents the estimated weights from quantile g-computation, reflecting the relative contribution and direction of each exposure in relation to eGFR. Positive weights (gray bars) indicate exposures associated with higher eGFR, while negative weights (black bars) reflect associations with lower eGFR. The weights sum to 1 within each direction, allowing interpretation of the most influential contributors.

Among the exposures, Mn had the strongest positive weight, eGFR, followed by o-xylene and Fe. In contrast, Cd contributed the most negatively to eGFR, followed by Se and Hg. Other exposures, including PFOS, Pb, PFOA, and m-/p-xylene, had minimal positive and negative weights.

### 3.5. Weighted Quantile Sum Regression (WQSR)

**Figure 8:**
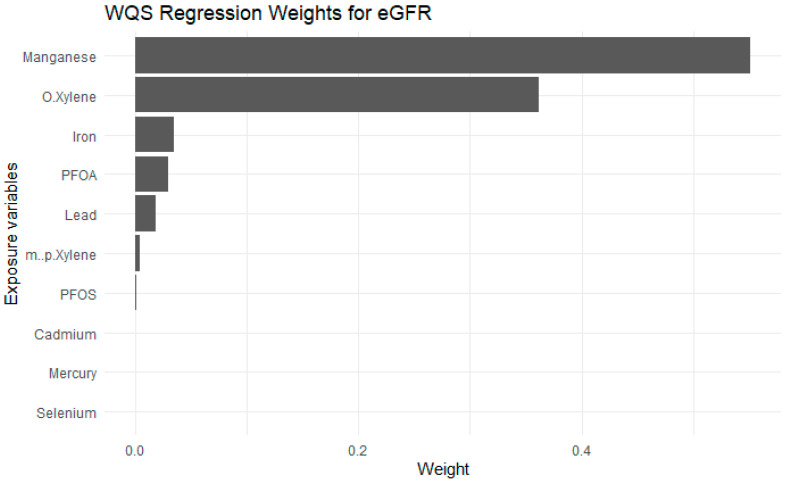
Weighted Quantile Sum Regression results of Exposures on eGFR. Adjusted for diabetes, alcohol use, smoking, income, gender, education, age, BMI and race/ethnicity. PFOS: perfluorooctanesulfonic acid, PFOA: perfluorooctanoic acid, eGFR: estimated glomerular filtration rate.

Figure 8 presents WQSR results, indicating the relative contribution of each exposure to the overall association with estimated glomerular filtration rate eGFR. The weights represent the proportionate influence of each exposure within the positive direction of the mixture effect and sum to 1. Among all exposures, Mn and o-xylene were the highest contributors, accounting for the largest weights. Fe, PFOA, Pb and m-/p-xylene also contributed moderately. In contrast, exposures such as PFOS, Cd, Hg, and Se contributed minimally.

## 4. Discussion

In this study, we explored the associations between a mixture of environmental exposures, including metals, essential elements, VOCs, and PFAS, with kidney function as measured by eGFR. eGFR is a key clinical biomarker for assessing renal function, with higher values indicating better glomerular filtration and overall kidney health [46]. In contrast, lower eGFR values suggest impaired kidney function, which may result from nephron damage caused by various factors, including environmental exposures [46]. We investigated these complex relationships between the exposure variables using advanced statistical models, including BKMR, quantile g-computation, and WQSR. The results highlight the complex, non-linear, non-additive, and potentially synergistic and antagonistic interactions among the exposures.

Spearman rank correlation analysis indicated substantial intercorrelations among metals, VOCs, and PFAS. In contrast, eGFR showed negative associations with these exposures, reflecting an inverse pattern between kidney function and increasing contaminant levels. Mn showed a positive correlation among other essential elements and weak correlations with eGFR, which suggests their positive effect on increased eGFR value, highlighting their potential for promoting better glomerular filtration and overall kidney function.

To better capture the complexity of the exposure–outcome relationship, we examined the PIPs generated from the BKMR model. The individual-variable PIPs indicated that Mn, Cd, Se, and PFOS had the highest PIPs, suggesting these exposures have the greatest relative importance in explaining variation in the eGFR outcome.

When evaluating group-level PIPs and conditional PIPs, the results highlighted o-xylene, PFOS, Cd, and Mn as the most influential within their respective exposure groups. This pattern implies that although entire groups of exposures contribute to the outcome, the posterior evidence points to specific chemicals within each group as the primary drivers of the observed associations with eGFR.

The univariate exposure–response analyses revealed that many of the exposures demonstrated non-linear associations with eGFR. For instance, Cd showed a U-shaped curve, while PFOS and Pb exhibited inverted U-shaped (∩-shaped) relationships. These patterns suggest that the biological response to these exposures varies across the exposure spectrum, with potentially protective or neutral effects at lower doses and harmful effects emerging at higher levels [47]. Mn displayed a strong positive association with increasing eGFR, indicating a potential protective effect on kidney function. In contrast, Hg, PFOA, Se, and m-/p-xylene showed relatively flat exposure–response curves, implying minimal or uncertain effects on eGFR within the observed exposure range. Consistent with these findings, the bivariate exposure–response functions also indicated similar trends, supporting the notion of complex interactions among exposure variables [47]. Collectively, these results suggest that environmental pollutants may contribute to adverse kidney health outcomes through dose-dependent and non-linear mechanisms. The observed patterns underscore the limitations of traditional linear models in capturing such nuanced relationships and highlight the importance of using flexible modeling approaches to better understand the health effects of environmental mixtures [48].

The result from the single variable effects of toxic metals, VOCs, PFAS, and essential elements on eGFR showed that Mn and Fe demonstrate a positive relationship with eGFR across all quantiles. This suggests that essential elements may have a positive effect on kidney function. In contrast, Cd had a strong negative relationship with eGFR, suggesting a detrimental effect of increased Cd exposure on kidney function, consistent with Cd’s known nephrotoxicity. The negative association of Se may reflect toxicity at higher exposure levels, despite its essential biological roles. Hg, Pb, PFOS, PFAO, m-/p-Xylene and o-Xylene showed weak negative to negligible association across quantiles suggesting little to no effect on eGFR within the exposure range. The weak negative associations observed for Hg, Pb, PFOS, PFOA, m-/p-xylene, and o-xylene with eGFR may be attributed to the relatively low to moderate exposure levels in the study population, which might not have been sufficient to cause measurable renal impairment. Additionally, the presence of protective elements such as Mn and Fe could have counteracted or masked the weaker nephrotoxic effects of these compounds. Findings from a similar previous U.S.-based NHANES study indicated that low blood concentrations of Cd and Pb were associated with higher odds of eGFR while low blood levels of Hg were associated with lower odds of eGFR, suggesting potential threshold effects or even adaptive mechanisms at lower exposures [20]. Another similar study also reported that higher erythrocyte Pb and Cd levels were significantly associated with increased risk of end-stage renal disease, while erythrocyte Hg showed a negative association, indicating a possible protective effect at low levels [49]. These results highlight the inherent complexity in understanding how environmental exposures affect kidney function, especially when multiple toxicants and essential elements interact and exert opposing physiological effects at the same time.

The results of the single-variable interaction analysis indicated that Fe and Mn exhibited the strongest interaction effects. These single-variable interaction terms were derived by comparing the effect of each exposure when all other exposures were fixed at the 75th percentile versus the 25th percentile. Examining how a single exposure interacts with all other exposures is particularly important in the context of environmental mixtures and the exposome, where no chemical acts in isolation. Such interactions can reveal whether the health impact of one exposure is amplified or attenuated depending on the levels of co-occurring exposures, thereby providing a more realistic assessment of risk than models that treat exposures independently. In the case of eGFR, these findings suggest that the burden of Fe and Mn may not operate additively but instead depends on the broader mixture environment. Since eGFR pathophysiology involves multiple pathways, including oxidative stress, inflammation, and impaired metal handling, the observed interactions highlight that susceptibility to eGFR may arise not only from elevated levels of individual metals but also from their combined effects with other co-exposures.

The overall exposure–response effect on eGFR levels showed a positive dose–response relationship between the exposure and kidney function. At lower exposure levels, the association appears neutral or slightly negative, but as exposure increases beyond the median, the effect becomes more clearly positive.

The results from the quantile g-comp and WQSR added further insight into the key drivers of eGFR between the exposures and the health outcome. The quantile g-computation analysis highlights the complex relationships between the exposures and kidney function by showing how these compounds contribute synergistically or antagonistically to eGFR changes [50]. Mn, Fe, and o-xylene exhibited positive weights, suggesting they may play a potentially protective or supportive role in maintaining kidney function within certain exposure ranges. In contrast, Cd, Hg, and Se had negative weights, indicating their adverse effects on eGFR and possible contributions to kidney impairment, particularly at higher concentrations [2]. Interestingly, PFOA, PFOS, and m-/p-xylene had negligible weights, suggesting they exert minimal direct effects on kidney function when considered individually. However, these compounds may still interact with other exposures in the mixture, producing either synergistic effects where combined exposures amplify kidney damage or antagonistic effects, where one compound reduces or offsets the harmful impact of another [51]. Unlike traditional regression models that assess exposures individually, WQSR creates a composite index of the exposures, assigning weights to each based on their contribution to the overall mixture effect. These weights sum to one and indicate the proportional influence of each exposure within the mixture, allowing us to identify the key drivers of the observed effects [52].

In this study, the WQSR results highlighted that Mn had the largest positive weights, suggesting it is the dominant contributor to improved eGFR within the observed exposure range. This aligns with their known biological roles of Mn, which is involved in antioxidant defense and metabolic regulation that may support kidney function [53]. Fe also showed a modest positive weight, reinforcing its potential protective effect when maintained within optimal physiological levels. On the other hand, exposures such as Cd, Hg, and Se contributed minimally in the WQSR model compared to quantile g-computation findings, likely due to their weaker independent associations or overlapping variance explained by more influential exposures. Similarly, PFOS, PFOA, and m-/p-xylene received very small weights, indicating limited individual impact on eGFR when considering the broader exposure mixture. These findings suggest that toxic effects may be masked when protective exposures dominate the overall index, or that the harmful impacts of certain toxicants emerge only at higher exposure concentrations not prevalent in the study population. These results are supported by previous studies demonstrating the importance of our study variables for kidney health. One study found that lower dietary Mn intake was associated with a higher risk of incident eGFR among adults with normal kidney function, suggesting that Mn deficiency may increase eGFR susceptibility [54]. Similarly, another study involving 7248 adults with diabetes from the UK Biobank showed that higher dietary Mn intake was linked to a lower risk of new-onset eGFR, particularly in individuals with poor glycemic control [55]. Conversely, prior evidence from the NHANES study (1999–2020, 55,677 participants) demonstrated that low-level Cd and Pb exposures were significantly associated with higher odds of eGFR, even after adjusting for demographics, comorbidities, and co-exposures. Specifically, individuals in the highest quartiles of CdCd and Pb had substantially elevated eGFR risk, with odds ratios ranging from 1.79 to 5.54 across adjusted models. Interestingly, Hg exposure was associated with lower eGFR odds at low-to-moderate levels, suggesting a possible threshold or biphasic effect. These findings support our observation that Cd and Pb are important contributors to renal dysfunction, whereas Mn and possibly trace Hg may play protective roles under certain conditions [20]. A recent NHANES-based study (2017–2018) supports our findings, showing that combined exposure to Cd, Hg, Pb, PFOA, and PFOS is linked to eGFR through non-linear and biphasic relationships. Using BKMR and logistic regression, the study identified Cd and Hg as the strongest contributors, while PFAS compounds and Pb had smaller effects. These results reinforce our findings that Cd and Hg are key drivers of kidney dysfunction and highlight the importance of using advanced statistical approaches to capture complex exposure interactions that traditional models may miss [17].

Given that our study also examined VOCs, it is important to compare our findings with prior research. A cross-sectional study of TFT-LCD industry workers investigated workplace VOC exposures and kidney dysfunction risk. Ethanol, acetone, isopropyl alcohol, and propylene glycol monomethyl ether acetate (PGMEA) were identified as the dominant VOCs. Array workers exhibited a 3.21- to 3.84-fold higher risk of kidney dysfunction compared to other workers, and those with cumulative exposures to isopropyl alcohol, PGMEA, and related compounds ≥324 ppb-year had a significantly elevated eGFR risk (adjusted OR = 3.41; 95% CI: 1.14–10.17). These findings demonstrate that chronic VOC exposure particularly solvents commonly used in manufacturing can contribute substantially to kidney dysfunction [56]. In our study, o-xylene showed a positive association with eGFR at lower exposure levels, suggesting possible threshold-dependent or biphasic effects, where low exposure may be neutral or protective but high cumulative levels increase nephrotoxicity.

The observed associations are biologically plausible given the well-established renal toxicity of several components in the mixture. The kidney is a primary site of accumulation and excretion for metals and other persistent compounds, rendering it particularly susceptible to oxidative and inflammatory injury. Lead and cadmium disrupt proximal tubular function, increase lipid peroxidation, and impair mitochondrial activity, whereas mercury interferes with thiol homeostasis and antioxidant defense. Per- and polyfluoroalkyl substances (PFAS), such as PFOS and PFOA, influence kidney function through peroxisome proliferator-activated receptor (PPAR) signaling, altered lipid metabolism, and tubular reabsorption pathways. Volatile organic compounds, including xylenes, can further promote oxidative stress and inflammatory cascades that exacerbate renal injury. In contrast, essential elements such as selenium and manganese may play a compensatory or protective role through their involvement in antioxidant enzyme systems. The BKMR and WQSR models collectively highlight these complex, and in some cases opposing, effects within the exposure mixture, underscoring the relevance of multi-pollutant modeling frameworks for elucidating mechanistic pathways linking environmental exposures to impaired kidney function.

This study highlights important public health implications, highlighting the need to mitigate harmful environmental exposures while promoting kidney health through targeted nutritional interventions. Cd and Hg were identified as major contributors to reduced eGFR and increased eGFR risk, whereas Mn and Fe showed potential protective effects at certain exposure levels. Reducing exposure to toxic metals and VOCs should be a priority through stricter environmental regulations, improved water filtration, and enhanced occupational safety measures, particularly in high-risk industries [44]. At the same time, promoting adequate intake of essential elements like Mn and Fe through dietary interventions or supplementation can help mitigate the effects of toxic exposure, especially in vulnerable populations. Targeted strategies are also needed for low-income and minority communities, which often face disproportionate pollutant exposure. Routine screenings and community-based prevention programs can aid in early detection and reduce eGFR disparities.

**Strengths and Limitations**

One of the principal advantages of this study lies in its application of modern statistical approaches for analyzing complex environmental mixtures. The integration of BKMR, WQSR, and quantile g-computation enabled a nuanced examination of how multiple environmental exposures jointly relate to kidney function. These complementary models made it possible to disentangle both the individual and combined influences of essential elements and toxic metals, offering a clearer understanding of potential synergistic and antagonistic effects on renal health. However, several limitations should be noted. First, the cross-sectional design of the NHANES dataset limits the ability to establish causal relationships, and longitudinal studies are required to confirm these findings. Although major covariates such as age, sex, population group, body mass index (BMI), and socioeconomic status were included in the analysis, the possibility of residual confounding from unmeasured or inadequately captured variables remains. For example, although information on medication use, dietary intake, and additional environmental exposures (such as phthalates) is available within NHANES, these variables were not integrated into the current analysis to maintain methodological consistency and focus on a specific, well-characterized mixture of metals, PFAS, VOCs, and essential elements. Incorporating these additional variables would have introduced substantial model complexity and potential multicollinearity, limiting interpretability within the Bayesian mixture framework. Nevertheless, these factors may influence kidney function through metabolic, inflammatory, or toxicokinetic pathways, and their potential role warrants further investigation in future studies designed to explicitly evaluate these broader exposure domains.

Lastly, although NHANES data was collected in the U.S. population, the results may not be directly generalizable to populations in regions with different environmental conditions, occupational exposures, or dietary habits.

**Future Research Directions**

Future research should employ longitudinal cohort designs to better elucidate temporal patterns and clarify causal linkages between environmental exposures and kidney function outcomes. Additionally, mechanistic studies exploring the biological pathways underlying synergistic and antagonistic interactions among metals, PFAS, and VOCs are warranted. Incorporating multi-omics approaches, such as metabolomics and proteomics, could provide deeper insights into how these exposures influence kidney health at the molecular level. Finally, expanding studies to diverse populations and geographic regions would improve the generalizability of findings.

## 5. Conclusions

This study provides evidence that environmental exposures, including metals, essential elements, VOCs, and PFAS, have complex, non-linear, and interactive effects on kidney function as measured by eGFR. We found that protective elements such as Mn and, to a lesser extent, Fe were positively associated with better kidney function, whereas toxicants like Cd and Pb demonstrated strong negative associations, indicating significant nephrotoxic potential. Interestingly, o-xylene exhibited positive associations at lower exposure levels, suggesting a possible threshold-dependent or biphasic effect, where low doses may be neutral or protective but higher cumulative exposures increase risk. In contrast, PFAS compounds and certain VOCs displayed negligible direct effects within the exposure range, but their potential to act synergistically or antagonistically within mixtures remains relevant. The integration of advanced statistical models BKMR, quantile g-computation, and WQSR allowed us to capture these non-additive and dose-dependent dynamics, offering a more realistic representation of real-world exposure scenarios. Collectively, our results emphasize the value of analyzing for environmental mixtures, rather than single exposures, when assessing kidney health risks and developing targeted public health interventions.

## Figures and Tables

**Table 1:** (a) Descriptive statistics of Exposure and Outcome Variables, (b) Demographic Characteristics of Study Participants.

(**a**)						
**Variable**		**Mean (SD)**	**Minimum**		**Maximum**	
Lead (ng/mL)		1.06 (1.35)	0.00		42.00	
Cadmium (ng/mL)		0.25 (0.59)	0.00		13.00	
Mercury (ng/mL)		1.07 (2.33)	0.00		64.00	
PFOS (ng/mL)		6.51 (7.74)	0.14		104.90	
PFOA (ng/mL)		1.71 (1.82)	0.14		52.87	
O-Xylene (ng/mL)		0.08 (2.27)	0.02		119.00	
m-/p-Xylene (ng/mL)		0.36 (12.30)	0.02		649.00	
Selenium (ng/mL)		186.30 (26.34)	85.00		454.00	
Manganese (ng/mL)		10.32 (3.77)	2.00		52.00	
Iron (ng/mL)		87.28 (36.62)	10.00		476.00	
eGFR (mL/min/1.73 m^2^)		98.63 (35.00)	3.44		313.44	
(**b**)						
**Variables**	**Description**	**Frequency**	**Mean**	**Percentage**	**95% CI**	
Gender (yrs)		9254				
	Male	4557	37.43	49.24	36.38	38.48
	Female	4697	39.38	50.76	38.12	40.64
Ethnicity		9254				
	Mexican American	1367		14.77	27.50	31.13
	Other Hispanic	820		8.86	31.77	35.60
	Non-Hispanic White	3150		34.04	40.00	43.34
	Non-Hispanic Black	2115		22.85	34.68	36.98
	Non-Hispanic Asian	1168		12.62	35.93	39.93
	Other Races—Including Multi-Racial	634		6.85	29.59	37.36
BMI	Male	8878	27.45		26.91	28.00
	Female	8931	27.45		27.21	28.55
Alcohol Use		5130				
	Yes	4545		88.60		
	No	585		11.49		

PFOS: perfluorooctanesulfonic acid, PFOA: perfluorooctanoic acid, eGFR: estimated glomerular filtration rate. Body mass index (BMI).

**Table 2:** Posterior Inclusion Probabilities for the influence of toxic metals and essential elements on eGFR.

Exposure Variables	PIP
O-Xylene	0.7224
m-/p-Xylene	0.5392
PFOA	0.2840
PFOS	1.0000
Lead	0.4880
Cadmium	1.0000
Mercury	0.0904
Selenium	1.0000
Manganese	1.0000
Iron	0.9852

PFOS: perfluorooctanesulfonic acid, PFOA: perfluorooctanoic acid, PIP: Posterior Inclusion Probability.

**Table 3:** Hierarchical BKMR results for eGFR. PFOS: perfluorooctanesulfonic acid, PFOA: perfluorooctanoic acid, eGFR: estimated glomerular filtration rate.

Exposure Variables	Group	Group PIP	Cond PIP
O-Xylene	1	0.982	0.8195519
m-/p-Xylene	1	0.982	0.1804481
PFOA	2	1.000	0.0000000
PFOS	2	1.000	1.0000000
Lead	3	1.000	0.0000000
Cadmium	3	1.000	1.0000000
Mercury	3	1.000	0.0000000
Selenium	4	1.000	0.0000000
Manganese	4	1.000	1.0000000
Iron	4	1.000	0.0000000

## Data Availability

The NHANES dataset is publicly available online, accessible at https://wwwn.cdc.gov/nchs/nhanes/continuousnhanes/overview.aspx?BeginYear=2017 (accessed on 12 September 2025).
